# The Impact of a Preterm Baby Arrival in a Family: A Descriptive Cross-Sectional Pilot Study

**DOI:** 10.3390/jcm10194494

**Published:** 2021-09-29

**Authors:** María Jiménez-Palomares, María Fernández-Rejano, Elisa María Garrido-Ardila, Jesús Montanero-Fernández, Petronila Oliva-Ruiz, Juan Rodríguez-Mansilla

**Affiliations:** 1 ADOLOR Research Group, Department of Medical-Surgical Therapy, Medicine Faculty, Extremadura University, 06006 Badajoz, Spain; mariajp@unex.es (M.J.-P.); jrodman@unex.es (J.R.-M.); 2 Terapeuta Ocupacional, Guadalete Alzheimer’s Residence and Day Center, 11500 Cádiz, Spain; mariafdezterapeutaocupacional@gmail.com; 3 Mathematics Department, Medicine Faculty, Extremadura University, 06006 Badajoz, Spain; jmf@unex.es; 4 Nursing and Physiotherapy Department, Nursing and Physiotherapy Faculty, Cadiz University, 11009 Cádiz, Spain; petronila.oliva@uca.es

**Keywords:** preterm infant, family impact, neonatal intensive care unit, occupational therapy

## Abstract

Background: The rate of premature births is increasing every day, with an estimated 15 million premature babies born worldwide each year. When a child is born prematurely, he or she is transferred to a Neonatal Intensive Care Unit (NICU), requiring special care on an ongoing basis. The admission of the newborn to these units can negatively affect the family routine as it generates changes and requires adaptation to new roles. Objectives: The objective of the present study was to understand the effect of the arrival of a premature baby on the family, based on the parents’ perception. Methods: A cross-sectional descriptive observational study conducted by means of a self-administered online ad-hoc questionnaire which collected information related to the situation of the relatives of premature infants in the region of Extremadura (Spain). The questionnaire consisted of a total of 35 questions, divided into three sections: ‘family environment’, ‘stay in hospital’ and ‘return home’. Results: Among the 53 responses obtained from fathers and mothers, 44 were from mothers. 53.6% of the respondents felt a delay in the acquisition of their parental role and 86.8% were afraid for their baby. During hospital stay, most of the parents had to modify their routines (94.3%), 69.8% suffered from sleep disturbances, 84.9% changed their eating habits and 88.5% referred to loss of time for themselves. Once at home, the time it took to recover their family normality ranged from 4 to 11 months, while 84.9% of respondents neglected their personal appearance and more than half had to give up or reduce their working hours. Conclusion: The arrival of a premature baby has a strong impact on the parents’ family environment, altering their daily routines and occupations both in hospital and at home. If preterm care programmes take into account these possible occupational imbalances, it will not only meet the needs of the parents but also provide family-centred care.

## 1. Introduction

An estimated 15 million premature babies are born worldwide each year, more than one in ten births [1,2,3]. In Spain, the number of preterm births in 2016 was 27,177, which represents 6.62% of all births in our country [4]. A preterm baby is defined as a child born at less than 37 weeks of pregnancy or 259 days post-conception, while a full-term delivery is considered when the birth is between 37 and 40 weeks of gestation [1,5]. Depending on the gestational age, preterm babies can be classified as moderate to late preterm (32–37 weeks), very preterm (28–32 weeks) and extremely preterm (less than 28 weeks) [1]. 

When a baby is born preterm, its hospitalisation in the Neonatal Intensive Care Unit can negatively affect the family organisation as it imposes changes in the daily routine of the newborn’s parents and requires adaptation to the new roles. Giving birth to a premature or sick baby is a stressful event for parents [6,7,8]. When the baby goes home after discharge from hospital, the support of the health care team comes to an end. The team was focused on stabilising the family during the period of hospitalisation and to facilitate the transition home. Therefore, the end of this professional support can lead to a feeling of emptiness in the family that can generate internal conflict and overload during their adjustment to life at home [9,10,11,12]. Discharge from hospital of preterm infants can thus be a critical event for parents that requires significant support from healthcare professionals. About 20% of preterm infants require specialized follow-up and home care [13,14]. This adds to the difficulty of release from work activities which constitutes an obstacle to the parent’s stay with the preterm infant. This will continuously interfere with the process of bonding and has an impact on the quality of interactions with the child, and may be considered a predictor of the development of the child’s cognitive ability [9,10,11].

Developmental centred care (DCC) was created in neonatal units with the main objective of promoting neurological and emotional development of the new-born and at the same time reducing the stress and burden of suffering for the relatives of the preterm infant [15]. When a preterm baby is born, it is normally admitted to the Neonatal Intensive Care Unit, a hospital unit in which care is provided to newborns with life-threatening or medical-surgical pathologies. The baby’s care is the responsibility of a paediatrician and other specialised doctors as the baby requires special care on an ongoing basis [16,17]. In general, admission to the neonatal unit aims to provide support for three basic vital functions: temperature control, breathing and feeding [9,16,18]. The preterm baby may be exposed to different external factors in the hospital such as noise from the technical equipment, noise caused by human movements and handling of material, strong luminosity, and so on. This environment may cause stressful situations and DDC has a key role in helping families and babies through hospitalisation [15]. Good health in early childhood contributes to appropriate brain development and to the achievement of a wide range of skills and learning abilities. Besides, there is evidence of a strong association between early adversities and environmental influences and a wide range of health-threatening behaviours in adulthood [19].

Interventions have almost always been linked to the baby itself, often forgetting the main caregivers. Many studies in recent years have focused on how mothers feel during the hospital stay, with many of them centering on the emotional aspects (fears, expectations, doubts, and so on) [6,7,8,20]. The presence of the parents and their participation in the care of the baby is essential to reduce stress and provide optimal care for both the premature or sick baby and the family [20].

Based on all of this, we considered that it was necessary to know the depth of the effect that the arrival of a premature baby has on the family, not only during the hospital stay but also during the whole process, until the return home. Being able to detect how daily occupations of the parents are affected (activities of daily living, leisure, play, work, education and social participation) can provide a better understanding of how to approach the whole intervention for the preterm infant. Consequently, it will help professionals to provide appropriate and effective family-centred care. Therefore, the objective of the present study was to understand the effect of the arrival of a premature baby on the family, based on the parents’ perception.

## 2. Materials and Methods

### 2.1. Study Design

This is an observational, descriptive, cross-sectional study carried out on a sample of parents of preterm infants in the region of Extremadura (Spain). The data were obtained through an ad-hoc on-line data questionnaire using the non-probabilistic snowball sampling method.

### 2.2. Ethical Aspects

All the ethical considerations and requirements mentioned in the Helsinki declaration [21] and the Data Protection Law [22] were met. The study was conducted under approval, responsibility and code of ethics of the “ADOLOR” research group with cataloguing code CTS048 which is registered in the section of Science, Technology, Innovation and University of the Ministry of Economy, Science and Digital Agenda of the regional government of Extremadura. Besides, the subjects included in the study signed the informed consent form to participate in the research.

### 2.3. Participants

The target population were parents of preterm infants who had gone through a hospitalisation period immediately after the birth of their baby. The association of fathers and mothers of preterm babies of Extremadura (APREMEX—Asociación de Padres y Madres de Niños Prematuros de Extremadura) and the association of parents of preterm babies (APREM—Asociación de Padres de Niños Prematuros) were contacted for this study and invited to collaborate. The inclusion criteria established to participate in the study were: parents of a premature baby, parents living in the region of Extremadura (Spain) and to give the informed consent to participate in the research. The exclusion criteria were as follows: date of birth of the premature child prior to the 31 May 2010 (i.e., children over the age of ten) and not being able to read and write in Spanish. The recruitment of participants was carried out during the months of April and May 2020.

### 2.4. Data Collection

In order to obtain the research data, an online ad-hoc questionnaire was used. It was developed by the authors of the study based on the most recent literature on the study subject. Databases such as Scielo and Medline, scientific search engines such as Pubmed and manual searches were consulted.

The self-administered questionnaire had a total of 35 questions divided into three distinct blocks. Before answering the first block, information about the study, the use and protection of data and the informed consent to participate in the research was provided. 

The first section, “family environment”, included 10 questions to collect the socio-emotional, socio-family and economic outcomes.

The second section, “stay in hospital”, collects information on the emotional stability of the parents, the routines, the activities of daily living, and their roles (questions 11–26).

The third section, “return home”, which corresponds to questions 27 to 35, continues to evaluate emotional variables, routines, roles, and adequate fulfilment of activities of daily living, but referring to their natural environment (homes).

Each question in the questionnaire, with the exception of the first one, which refers to the informed consent and has only one response option “I accept”, is accompanied by a series of response options depending on the content of the question. The father or mother should mark a single response that most accurately reflects his/her current or past situation.

### 2.5. Procedure

All participants were informed that their participation in the study was anonymous and voluntary and that no incentives would be offered. The invitation to participate in the research was distributed by email and mobile phone along with the link to the online questionnaire. The questionnaire was available during the months of April and May 2020, after which time data collection was finished.

### 2.6. Data Analysis 

All responses were coded to ensure the anonymity of the participants and were analysed by staff external and independent to the study. All variables analysed are categorical, thus the results are presented as frequency distributions. Besides, the associations between the variables were analyzed by the χ^2^ test (provided that the validity conditions were met) and Pearson’s C (a meassure of association, also known as the contingency coefficient, whose maximun value is 0.70 for our design). Only significant associations with the main outcomes were reported, with their respective contingency coefficient. A Multiple Correspondence Analysis (MCA) was carried out in order to understand the relationship on the whole. A multiple binary logistic regression was applied with the aim of detecting counfunding variables. Correlations were considered significant at *p* < 0.05. SpSS version 26.0 was used for the statistical analysis.

Although the participants were aware of the purpose of the survey, it was completely anonymous, thus preserving the privacy and confidentiality of the responses.

## 3. Results

A total of 53 responses were obtained. The results are described according to the different sections of the questionnaire: family environment, hospital stay and return home.

### 3.1. Family Environment

Of the total sample (*n* = 53), 44 surveys corresponded to mothers and only 9 to fathers of preterm babies. Further, 73.8% of the total number of fathers and mothers resided at the time of the birth of their babies in a rural environment.

The family characteristics of the respondents are summarised in Table 1. This information corresponds to items 3 to 10 of the questionnaire.

### 3.2. Hospital Stay

**Figure 1:**
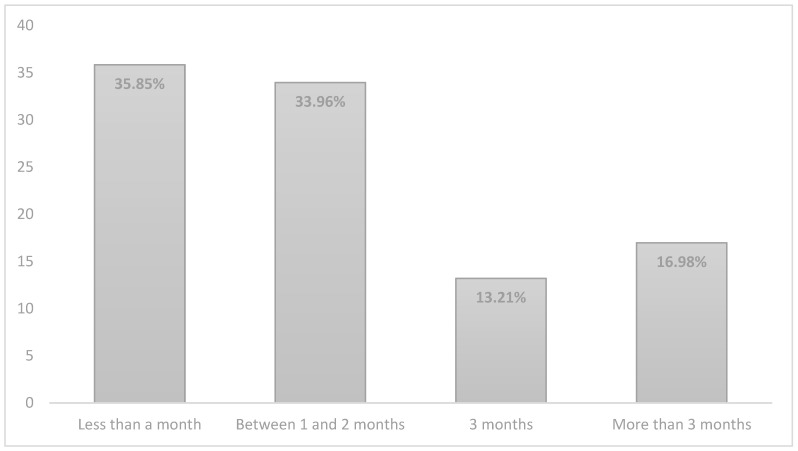
Length of stay of the babies at hospital.

Regarding the questions of the second block ‘hospital stay’, the results showed that the majority of parents (77.4%) had to stay at the hospital for less than a month. However, the average length of time that the children were hospitalised was between 1 and 2 months (Figure 1). This means that the baby had to stay in the hospital and the parents had to visit them.

A total of 86.8% of the parents were afraid for their child’s life. Most of them were not in their place of residence (77.4%). However, 68.2% were offered a place to stay in the city in which the hospital was located at no additional cost. The fact of having accommodation during the hospital stay facilitated continuous attendance at the health centre for 100% of the respondents.

Owing to the hospital environment and the prematurity of their children, 53.6% of the parents felt that the acquisition of their maternal/paternal role, which provides security and family cohesion, was delayed.

**Figure 2:**
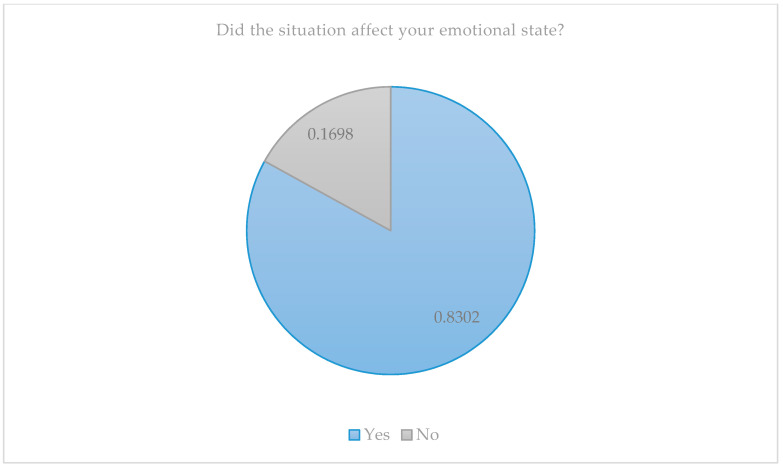
Impact of the situation on the parents’ emotional state.

When we delved deeper into the personal situation of the family members, we found that every situation experienced had a significant impact on their emotional state (Figure 2).

The impact on the parents’ daily life during the hospital stay is shown in Table 2. These data correspond to items 20 to 25 of the questionnaire. We can observe that their children’s hospitalisation implied that the parents had sleeping deficiencies, their food was not very healthy eating mainly fast food and they stopped spending time on leisure activities. They also had to modify their daily routines and even their concentration at work was affected. 

**Figure 3:**
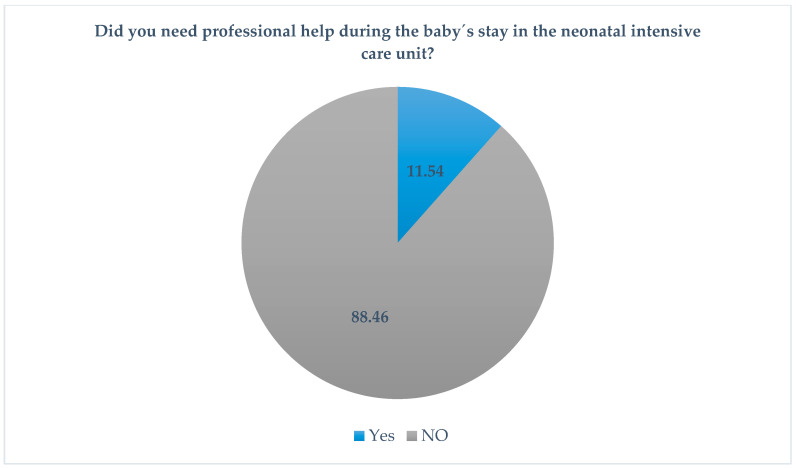
Professional help.

Despite all these changes in the daily routine of the parents, only six of the respondents sought professional help during their baby’s stay in the neonatal intensive care unit (Figure 3).

### 3.3. Return Home

On returning home, 69.8% of the parents were afraid that they would not be able to provide appropriate care to their baby without the help of professionals. However, they reported that after a few months this concern diminished. Moreover, 47.2% of the respondents reported that their children needed specialised care once they left hospital. This percentage corresponds to 25 out of 56 respondents.

Table 3 shows the changes in the parents’ daily lives once they were at home with their babies (questions 30 to 35 of the questionnaire). More than half of the parents found it difficult to resume their daily routines which took an estimated time of 4 to 11 months after the baby’s return home. The vast majority of the parents experienced a lack of attention to their personal image and a change in their personal relationships. However, all these changes did not affect the parents’ relationships with their partner. In relation to their working lives, the results indicated that half of the respondents had to stop working or to reduce their working hours.

**Figure 4:**
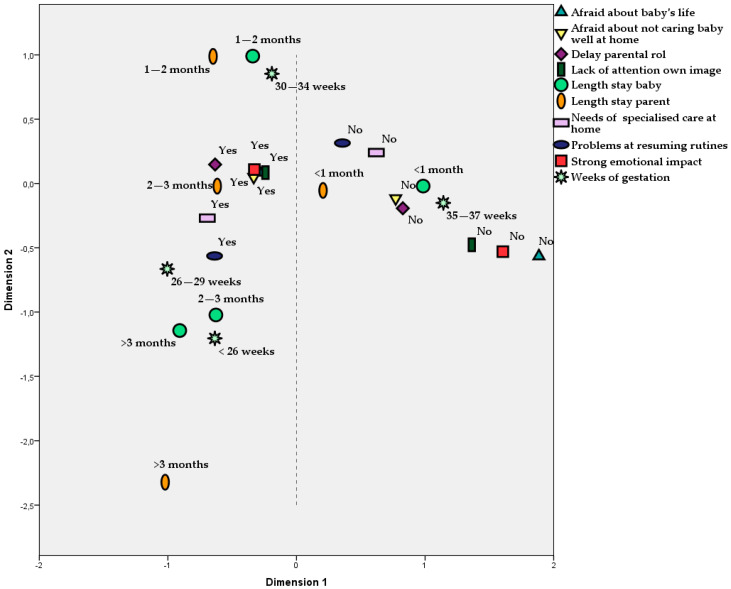
Multiple correspondence analysis for the main outcomes. This figure should be understood as follows: all the categories of the same outcome are represented by the same figure. If two categories of different outcomes are plotted closely, it means that they are positively associated, as occurs, for example, for String emotional impact = Yes and Lack of attention own image = Yes.

In the analysis of associations, strong correlations between ‘Weeks of gestation’ and ‘Length of the stay of babies at hospital’ (*p* < 0.001, Person’s C = 0.688) were found. In fact, these two variables could be considered practically the same. We also detected a strong correlation between ‘Weeks of gestation’ and ‘Impact on parents’ emotional state’ (*p* = 0.010, C = 0.423). Evidently, an association in the same way was found between ‘Length of the stay of babies’ and ‘Impact on parent’s emotional state’ (*p* = 0.003, C = 0.458). These results are illustrated in Figure 4. However, ‘Length of parent’s stay’ did not explain well their emotional state, as it was non-significant when it was included, together with ‘Length of babies’ stay’, in the same logistic regression model (*p* = 0.116 for parents and *p* = 0.006 for babies). 

‘Length of babies’ stay’ correlated in an obvious way with ‘Need of specialised care’ once they left the hospital (*p* < 0.001, C = 0.534). The outcomes that correlated significantly with ‘Impact on parents’ emotional state’ were as follows: Parents were afraid for their child’s life during hospitalization (*p* < 0.001, C = 0.492), Parents were afraid of not being able to provide appropriate care to their baby (*p* < 0.001, C = 0.424), Delay in acquisition of parental role (*p* = 0.022, C = 0.299), Parents could resume daily routines (*p* = 0.014, C = 0.324) and Parents show a lack of attention to their personal image (*p* < 0.001, C = 0.455). These are, according to our sample, the most evident demonstrations of the parents’ emotional impact.

Figure 4 summarises all of these correlations obtained with the MCA. The proximity between figures corresponds to a positive association. We can see, on one hand, that all negative answers are located on the right side of the plot, near to figures corresponding to 35–37 weeks of gestation and under one month of stay at the hospital. On the other hand, affirmative answers, corresponding to emotional impact, are very close to the left side, linked to gestation under 35 weeks and longer hospitalizations. 

## 4. Discussion

The aim of our study was to find out the effect of the arrival of a preterm baby on the family environment. Our results suggest that parents undergo major changes in their daily routines, roles (particularly parental roles), and occupations such as work and activities of daily living (feeding, sleeping and personal care), both during hospital stay and upon returning home.

Being able to know the impact on the parents’ family life is an important step in family care because, together with understanding mothers’ experiences, it enables us to recognise their needs and address them effectively [23].

The survey was completed by only 9 male parents out of a total of 53 participants. This fact has prevented us from being able to make a realistic comparison with the mothers’ responses in order to compare the feelings or situations of both groups. In this sense, the studies found in the literature focus only on one of the parents [3,24,25] without making an interrelation between them, as we do in our study.

The emotional impact on parents of the hospitalisation of a premature baby refers to the feeling of sadness, fear and separation stress [6,7,8,20,23,26,27,28]. This can make parents feel fragile and insecure about their child’s life. The lack of opportunities to interact affectionately with their baby can lead to a loss of connection [28]. The disruption of the mother and child bonding has a negative impact on mothers that can be aggravated by the clinical condition of the newborn [3,28,29]. These fears were reflected in the results of our study, which showed that 86.8% of the parents felt fear for their child’s life and half of them felt that the acquisition of their parental role was delayed. This coincides with other descriptive studies [26,27,28] with similar characteristics. In this sense, it is known that parental competence acts as a protective factor against stress in mothers and caregivers, as it directly influences parental practices in child-upbringing contexts [30].

Having routines and habits is the basis of people’s occupational balance. They can influence the state of health; therefore, everyone should manage them appropriately [31]. A person’s balance in the organisation of their daily activities contributes to a successful occupational performance. Likewise, occupational performance contributes to life satisfaction, and thus to the quality of life. Occupational balance is unstable; therefore, it is necessary to control the different situations that a person has to face in life in order to maintain that balance [32].

Having a baby hospitalised will alter the usual occupations and roles related to family life and work among others [33]. This statement is supported by the results of our research, as we found that during the hospital stay, 88.5% of parents reported a lack of time for themselves, having to modify their routines in 94.3% of cases. In addition to these changes, there was an alteration in the eating habits, with an increase in fast food intake. There was also a negative impact on the working life, with 90.2% of parents reporting a decrease in concentration while they were at work. We consider that it would be important to take into account these aspects in order to include them in the support schemes and programmes that hospitals provide to parents during their children’s stay in the neonatal intensive care units. A good occupational balance, adjusted to the capacities and needs of the person and related to their personal interests, satisfies the subject’s own needs and the expectations of the environment [34].

It is necessary not to neglect the needs of the parents as caregivers, because, as we have seen in 83% of parents, the situation of their child’s hospitalisation had a significant impact on their emotional state. Therefore, it is necessary that health professionals increase their awareness of the psychosocial needs of mothers with preterm babies [27]. It is known that the state of a mother’s mental health has a significant impact on the development of the relationship between the mother and her baby and on the mental and physical health of the baby [27,35].

As the results of our study indicate, the impact on the family after the arrival of the premature baby is not only during the hospitalisation stay. Once they arrive home the problems are still present. In particular, if one of the parents has to take the lead in the care of the baby adopting a nurse-like role, it can affect their family and social environment [13]. The baby’s discharge from the neonatal intensive care unit can be experienced as a moment of mixed feelings. Going home is a happy event for the parents but, at the same time, it is accompanied by anxiety and worry [36]. In our study, we could observe that 69.8% of the respondents had uncertainty at the time of going home. Moreover, the parents had medical worries as 47.2% of the children needed medical care once at home and they suffered changes in social relations. These data suggest that future studies need to focus on this stage as well, based on our findings, we have perceived that there is an inadequate family preparation for discharge, especially for the mother [24]. Confident parents who are prepared to take their baby home, with individualised information, guidance and practical experience in caring for their baby before discharge, may be of vital importance [36].

Social support is important to reduce the burden on parents and is also identified as a key element in reducing the incidence of perinatal mental disorders. In contrast to the study by Fowler et al. in 2019 [27], the participants of our study did report having social support. Further, 98.1% reported that they had a good family environment and 96.2% had the help of the other parent in the care of the baby. These data may explain why only 1.54% needed professional help. We have also been able to observe how short baby’s stays are associated with a lack of parents’ problems (in general) and long stays are associated with the presence of parents’s problems. In the same way, the results showed that long stays are associated with less weeks of gestation and the need for specialised care. Besides, parents’ problems appeared to be associated between them.

Most of the articles related to the research subject that were consulted [13,26,27,28,29] focus on the stress and feelings expressed by family members and caused by the arrival of their premature babies or those feelings experienced in the neonatal units. However, there are scarce studies in the literature that focus on the occupational changes experienced by parents [24]. In this sense, we believe that our results may serve to explore this topic in greater depth. Our study has shown that parents suffered from a lack of personal image, had a loss of social relationships, experienced a decrease in leisure activities, had changes in their routines, and showed a decrease in work performance.

These results can help health professionals to understand the reality experienced by parents of premature infants better and to be able to care for them in a more comprehensive way, both in the hospital stay and in their own environments. Being able to adapt to their needs, not only emotionally, but also in their daily lives, will facilitate the planning of realistic and family-centred care programmes. 

### 4.1. Limitations of the Study

One of the limitations of the study is that the parents had difficulty in remembering past experiences with their children. The time that passed since the birth of their premature baby may be the reason that some aspects of their experiences could have been difficult to recall accurately. However, there is scientific evidence that supports that experiences such as the arrival of a baby are ‘unforgettable events’ [37]. In addition, the babies’ development could have influenced the parents by having more positive or negative memories depending on whether their baby did well or not. Whether the difficulty to remember was associated or no to a worse baby growth or development is possible but not clear. Although we found in our sample an association between parent’s emotional impact and the need of specialised baby care at home (see Figure 4), it was not significant (*p* = 0.100, C = 0.220), which could be due to another limitation of the study: the sample size. The sample may be considered small and not representative of the entire population (*n* = 53). In general, and with this sample size, the χ^2^ test is able to detect a significant association only for an effect size (Cramer’s V) over 0.27, i.e., just for correlations commonly consider medium or large. So, we would need a bigger sample to find out weaker correlations. Nevertheless, our sample size is far superior to other similar descriptive studies [18,21,24,25,26] whose sample size ranges from 10 to 20 participants. The sample size, together with the lack of variability or excess of categories in some outcomes did not allow us to apply the χ2 test in many cases. For this reason, it would be convenient in future studies to limit the time that has elapsed since they had their premature baby, as developments in preterm care may suffer changes as time goes by. We also consider that it would be very interesting in future research to have a larger and more representative sample, to take into account the additional health problems besides prematurity that the babies may have, and to assess how hospital readmissions affect families.

The fact that the survey was done through an online questionnaire and by the snowball sampling method allowed us to reach a larger sample. However, it also made it more difficult for the researchers to control the sample.

On the other hand, it is important to highlight that in each country the conditions under which women give birth could be different. Each woman gives birth in different hospitals or settings which could have different health care protocols. In addition, health services and neonatal intensive care units’ policies may differ and could condition parents’ experiences. Therefore, these findings could not be extrapolated to populations in other countries. 

### 4.2. Clinical Implications

Although this is a qualitative study with no generalisable statistical findings, these results can be used to better understand the needs and difficulties that parents of preterm infants’ experience during hospitalisation and after discharge.

Knowing what impact the arrival of a premature baby has on the parents’ lives, particularly on their habits, routines and daily activities, will allow the development of family-centred strategies and interventions that can provide effective care for both the baby and the parents.

## 5. Conclusions

The arrival of a premature baby has a strong impact on the parents’ family environment, altering their daily routines and occupations both in the hospital and at home. If preterm care programmes take into account these possible occupational imbalances, it will not only meet the needs of the parents but also provide family-centered care.

## Figures and Tables

**Table 1:** Data related to the family environment.

ITEMS	CATEGORY	%
Support between both parents	Yes	96.2
	No	3.8
Parent’s working status	Both parents are employed	53.8
	Only the mother is employed	3.8
	Only the father is employed	42.3
Educational level	Secondary school (compulsory education)	28.3
	Further education	3.8
	Vocational training (high level/degree)	18.9
	University degree	34.0
	Vocational training (intermediate level/degree)	15.1
Socio-economic status	High	3.8
	Middle	81.1
	Low	15.1
Good family environment	Yes	98.1
	No	1.9
Problems during pregnancy	Yes	41.5
	No	58.5
Weeks of gestation	37–35 weeks of gestation	26.9
	34–30 weeks of gestation	42.3
	29–26 weeks of gestation	9.6
	Less than 26 weeks of gestation	21.2
Number of other children in the family	0	54.7
	1	39.6
	2	5.7

**Table 2:** Impact of the hospital stage on the parents’ lives.

Items	Options	%
Restorative sleep	Yes	30.2
	No	69.8
Lack of self-dedication	Yes	88.5
	No	11.5
Could carry out activities of your interests	Yes	13.2
	No	86.8
Feeding	Often have fast food	84.9
	Balanced diet	15.1
Had to modify routines	Yes	94.3
	No	5.7
Concentration at work	Yes	9.8
	No	90.2

**Table 3:** Impact on the parents’ lives once at home after discharge.

Items	Options	%
Resume daily routines	Yes	64.2
	No	35.8
Time to return to daily normality	1–3 months	29.4
	4–11 months	43.1
	More than 1 year	27.5
Neglect of personal image	Yes	84.9
	No	15.1
Left work or reduced working hours	Yes	55.8
	No	44.2
Change in personal relationships	Yes	92.5
	No	7.5
Relation with the partner was negatively affected	Yes	35.8
	No	64.2

## Data Availability

The data underlying this article cannot be shared publicly to maintain the privacy of individuals that participated in the study. The data will be shared on reasonable request to the corresponding author.
